# Autism and psychosis as diametrical disorders of embodiment

**DOI:** 10.1093/emph/eoz021

**Published:** 2019-07-15

**Authors:** Bernard Crespi, Natalie Dinsdale

**Affiliations:** 1 Department of Biological Sciences, Simon Fraser University, 8888 University Drive, Burnaby, BC, Canada; 2 Department of Psychology, University of Saskatchewan, Saskatoon, SK, Canada

**Keywords:** rubber hand illusion, embodied cognition, autism, schizophrenia, anorexia, interoception, predictive coding

## Abstract

Humans have evolved an elaborate system of self-consciousness, self-identity, self-agency, and self-embodiment that is grounded in specific neurological structures including an expanded insula. Instantiation of the bodily self has been most-extensively studied via the ‘rubber hand illusion’, whereby parallel stimulation of a hidden true hand, and a viewed false hand, leads to the felt belief that the false hand is one’s own. Autism and schizophrenia have both long been regarded as conditions centrally involving altered development of the self, but they have yet to be compared directly with regard to the self and embodiment. Here, we synthesize the embodied cognition literature for these and related conditions, and describe evidence that these two sets of disorders exhibit opposite susceptibilities from typical individuals to the rubber hand illusion: reduced on the autism spectrum and increased in schizophrenia and other psychotic-affective conditions. Moreover, the opposite illusion effects are mediated by a consilient set of associated phenomena, including empathy, interoception, anorexia risk and phenotypes, and patterns of genetic correlation. Taken together, these findings: (i) support the diametric model of autism and psychotic-affective disorders, (ii) implicate the adaptive human system of self-embodiment, and its neural bases, in neurodevelopmental disorders, and suggest new therapies and (iii) experimentally ground Bayesian predictive coding models with regard to autism compared with psychosis.

Lay summary: Humans have evolved a highly developed sense of self and perception of one’s own body. The ‘rubber hand illusion’ can be used to test individual variation in sense of self, relative to connection with others. We show that this illusion is reduced in autism spectrum disorders, and increased in psychotic and mood disorders. These findings have important implications for understanding and treatment of mental disorders.

We don’t see things as they are, we see things as we are Anaïs Nin ([1], p. 124)

## 1. INTRODUCTION

An evolutionary approach to understanding human psychiatric disorders compels determination of what cognitive adaptations have evolved along the human lineage, and how these traits become subject to effects of extreme development, trade-offs, mismatches and genetically based conflicts that lead to maladaptation. Among the most remarkable of human-evolved and human-elaborated psychological phenotypes are those associated with the self, which encompass self-awareness, spatial-temporal location, identity, agency and embodiment [2–5]. These semantic and psychological self-constructs are subserved by neural structures and networks that, over the past decade, have become sufficiently well understood for empirical analyses that connect neurological adaptations to psychiatric conditions, in the context of quantitative, predictive models of neural activity (e.g. [6]). What, then, are human disorders of the self, and how can they be analysed in such frameworks?

Two disorders come immediately to mind, from the etymology of their names. First, the term ‘autism’ derives from the Greek autos (ἀϝτός), for ‘self’. The core of this disorder, as originally described by Kanner [7], is extreme aloneness, and psychological separation between one’s self and others:

The outstanding ‘pathognomonic’, fundamental disorder is the children’s inability to relate themselves in the ordinary way to people and situations from the beginning of life. The parents refer to them … ‘like in a shell’… There is from the start an extreme autistic aloneness that, whenever possible, disregards, ignores, shuts out anything that comes to the child from the outside.

In modern terms, this ‘self’-oriented cognition of autism is reflected in reduced social and communicative interactions, less-developed social imagination, attention highly focused on non-social stimuli, repetitive self-related behaviors, and narrow non-social interests. Most generally, in terms of altered, human-elaborated adaptation, autism can thus be regarded as reflecting under-development of the ‘social brain’, the distributed set of neural systems dedicated to the acquisition, processing, and deployment of social information [8]; the non-social, isolated ‘self’ brain remains.

Second, the word ‘schizophrenia’ derives from the Greek for ‘split’ (σχίζειν) ‘mind’ (φρήν), referring to loss of unity and boundaries of the self. From Bleuler [9], one of the first to analyse this condition:

If disease is marked the personality loses its unity … one set of complexes dominates the personality for a time, while other groups of ideas or drives are ‘split off’ and seem either partially or completely impotent … Everything may seem different; one's own person as well as the external world … in a completely unclear manner so that the patient hardly knows how to orient himself either inwardly or outwardly …. The person loses his boundaries in time and space.

Kraepelin [10], perhaps the first ‘biological’ psychiatrist who studied schizophrenia, similarly believed that ‘dissolution’ of self-experience was a fundamental aspect of schizophrenia.

These descriptions of the self in schizophrenia manifest specifically and most clearly in Kurt Schneider’s ‘first-rank’ symptoms, most of which represent dysregulated components of the self [11]. Recent cognitive-psychological and phenomenological treatments of schizophrenia emphasize altered aspects of the self as central to this disorder, involving, for example, exaggerated self-consciousness, high levels of dissociation (feeling of detachment from one’s body), and loss of unitary embodiment [12–17]. In this context, schizophrenia represents one of a set of related psychotic and affective (mood) disorders, that also includes bipolar disorder, major depression and borderline personality disorder (among others); these conditions exhibit broadly overlapping risk factors and symptoms including alterations to the self, and grade into one another as well as into typical cognition [8].

In terms of dysregulated adaptation, many psychotic-affective disorder symptoms can be regarded as involving exaggeration of components of the social brain, most notably excessively social cognition and affect expressed in paranoia, social delusions, other social and self-related forms of hypermentalization, auditory hallucinations, mania, and social emotions (e.g. pride, guilt, embarrassment, shame) [18–21]. Most broadly, such social hyper-developments, especially though not exclusively in forms of psychosis, represent manifestations of the self merging and interacting more readily and deeply, in negative and pathological ways, with other social entities, both imagined and real; first-person accounts reflect this perspective, as well as dissociation, excessive self-consciousness [14] and depersonalization, all of which indicate expansive alterations to self**-**experience [22, 23].

Based on such observations among others, Crespi and Badcock [24] proposed that autism and psychotic-affective conditions represent diametric, ‘opposite’ disorders, with regard to their phenotypes, genetic and neurological bases, and their evolutionary grounding in self and social brain evolution and development [8, 25]. This hypothesis can be tested most directly through comparisons of individuals on the autism spectrum to individuals on the psychotic-affective spectrum (usually in the context of schizophrenia, with specific emphasis on psychosis), performing the same task that provides insights into self and social cognition. For example, a recent study [26] showed that levels of anomalous self-experience were substantially and significantly higher among subjects with Schizotypal Personality Disorder than those with Asperger syndrome, sufficiently so for useful clinical differentiation.

**Figure 1.**
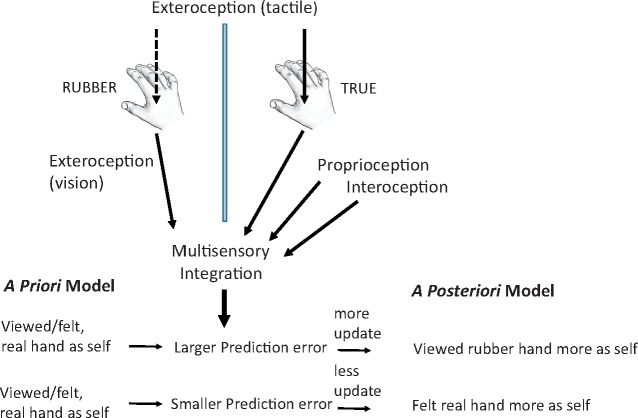
The rubber hand illusion and its predictive-coding-based explanation

A primary task used by psychologists and neurologists to quantify self-embodiment is the ‘rubber hand illusion’, a protocol that involves experimental manipulation of multisensory integration to measure the degree to which subjects can be induced to feel as though an artificial hand is their own (reviews in [2, 3]). By this method, subjects view a rubber hand that is adjacent to their own, hidden hand; both hands are then touched by the experimenter in the same spots, usually with a paintbrush (Fig. 1). Individuals then, to a variable degree, come to feel as though the rubber hand is a part of their own body, and their real hand can become ‘disowned’. These effects are usually quantified using some combination of three measures: (i) time until the illusion is felt; (ii) self-report of illusion intensity and (iii) self-location of one's ‘own’ hand in space, between the real and false hand (so-called proprioceptive drift). The latter two measures are postulated to be mediated by more or less independent neurophysiological and psychological effects [27], although they are significantly positively correlated with one another in some studies (e.g. [28]).

At the neurological level, the rubber hand illusion reliably activates the insula [29–36], as well as other brain regions that subserve multisensory integration from visual and tactile cues, such as premotor and parietal cortex [37, 38]. The insula in particular is commonly linked with the self, in the context of self-embodiment and self-awareness [29–32]. This region represents the brain's hub for interoception, the sensing and neural representation of internal physiological and emotional state, in contrast to exteroception as perception of external stimuli, and proprioception as sensing the position and movement of one's body.

At a cognitive level, the rubber hand illusion is typically interpreted in the context of a Bayesian, predictive coding framework of brain functioning, whereby *a priori*, higher-level, predictive neural models are continually compared for congruence with lower-level sensory inputs; differences between the two (called ‘prediction errors’, recognized as salient) are reconciled by changing either the model or the inputs [6, 39]. Here, the model of the rubber hand as not-self, based on proprioceptive and interoceptive stimuli, becomes incongruent with exteroceptive visual and sensory-touch information, leading to resolution through model updating by considering the rubber hand as one's own and the true hand as not-self (Fig. 1). The extent to which the illusion is felt depends on how the forms of sensory information are integrated, and on the precisions of their estimates, more-precise sensory-neural information and more-precise higher-level models, being afforded higher weight with regard to prediction error detection and model updating.

The rubber hand illusion is associated with insula function, and function of the regions that interact with this part of the brain, in the context of embodied cognition, with higher feeling and endorsement of the illusion associated with lower self-embodiment and weaker self-other boundaries [3, 40]. This protocol is especially useful for comparing autism spectrum and psychotic-affective spectrum conditions because its neurophysiological basis is interpretable in terms of predictive coding models; such models have also been developed and applied for understanding autism [41–43] and psychosis [44–46], but they have seldom been evaluated in terms of how the two disorders are related to one another [47]. The illusion is also especially valuable because its quantification and analysis are based on differences, which indicate altered function, rather than deficits, which are non-specific and subject to many diverse causes.

This paper represents a synthesis of the literature regarding the effects of the rubber hand illusion in autism spectrum and psychotic-affective spectrum conditions, to evaluate the hypothesis that these disorders represent opposites with regard to embodied cognition and traits directly associated with it. This analysis also bears directly on the nature of alterations to predictive coding in autism and psychosis; as such, it may provide general insights into neural functioning in these disorders and in typical cognition.

## 2. METHODS

The literature was searched exhaustively for studies that used the rubber hand illusion in relation to autism spectrum conditions (both clinical disorders and non-clinical autism traits), and psychotic-affective conditions (including schizophrenia, bipolar disorder, depression, non-clinical schizotypal traits, borderline personality disorder and frontotemporal dementia [48–51]), using PubMed, Web of Science and Google Scholar. Eating disorders were also included, given their links with psychotic-affective conditions (described below), as were studies that involved administration of drugs (ketamine or amphetamine) that can induce psychotic states, and thus serve as pharmacological proxies of psychotic conditions. Only studies that used the classical, original rubber hand illusion or a simple reach-to-grasp extension of it, and measured some combination of time to feel the illusion, subjective illusion endorsement and proprioceptive drift, were included.

Four additional sources of information were integrated into this synthesis: (i) studies of interoceptive awareness, measured as heartbeat perception accuracy, because this variable has strong, inverse links to rubber hand illusion susceptibility [3, 52], and provides a measure of sensorineural interoceptive precision; (ii) studies of oxytocin in relation to the rubber hand illusion, because this neurohormone has been associated with insula activation, interoception, empathy, and autism and psychotic-affective conditions [53–55]; (iii) studies of empathy (self-report, and other measures) in relation to the rubber hand illusion, and in relation to the other variables; and (iv) studies that measured genetic correlations of autism or schizophrenia with empathy or eating disorders (or other disorders), because such studies provide insights into the overlapping or independent genetic bases of variables associated with the rubber hand illusion. Genetic correlations are usually the result of pleiotropy, whereby, integrated across the genome, alleles affect multiple traits in the same or opposite directions.

The analysis thus represents a narrative analysis focused on (i) evaluating the hypothesis that autism and psychotic-affective conditions show similar or opposite patterns with regard to rubber hand illusion susceptibility, and (ii) determining the degree to which the other relevant variables can be integrated into this evaluation, in a consilient manner consistent across the patterns of pairwise variable associations for which data are available.

## 3. RESULTS

### 3.1. Associations of predictor variables with rubber hand illusion susceptibility

All five studies involving the autism spectrum, which included six analyses, demonstrated slower or reduced effects of the rubber hand illusion in subjects with autism (three analyses), or in subjects with higher non-clinical autism spectrum scores (three analyses) (Table 1). In contrast, all 11 studies of psychotic-affective conditions, which included 12 analyses, showed faster or stronger effects of the illusion associated with these conditions, for at least 1 test: 6 of these analyses compared schizophrenia subjects versus controls, 4 analysed levels of schizotypy, psychoticism or psychosis proneness, one analysed borderline personality subjects, and one analysed subjects with c9orf72-mutation induced frontotemporal dementia. Illusion strength was linked with positive-schizophrenia symptoms in four of the six analyses that addressed this comparison [61, 62, 66, 67]. In the single study of borderline personality subjects, levels of dissociation were also positively associated with self-report illusion strength. Treatment with the psychosis-mimetic drugs ketamine or amphetamine, and chronic ketamine use (without administration at the time of testing) were also associated with a stronger rubber hand illusion, in all three of these studies.

**Table 1.** Evidence regarding susceptibility to rubber hand illusion in relation to the autism spectrum, the psychotic-affective spectrum, eating disorders, oxytocin and empathy

Group(s) studied	Latency to feel illusion	Sense of ownership, self-report	Proprioceptive drift	Citation	Comments
Autism spectrum					
ASD subjects versus controls	Not tested	No effect	Lower in subjects with ASD	[56]	
ASD subjects versus controls	Slower in ASD subjects	No effect	No effect	[57]	Also tested effects of empathy; see table entry below
Typical subjects varying in AQ scores	Not tested	No effect	Lower in subjects with higher AQ score	[58]	Also found reduced illusion effects in reach-to-grasp movements for typical subjects with high AQ scores
ASD versus controls, and typical individuals varying in AQ scores	No effects	No effects	No effects	[59]	Found reduced illusion effects in reach-to-grasp movements, for subjects with autism and for typical subjects with high AQ scores
Typical subjects varying in AQ scores	Not tested	Lower in subjects with higher AQ score	No effects	[60]	Also analysed serum oxytocin levels; see table entry below
Psychotic-affective spectrum					
Schizophrenia subjects versus controls	Faster in schizophrenia subjects	Higher in schizophrenia subjects	Not tested	[61]	Illusion strength positively associated with positive but not negative symptoms
Typical subjects scored for metrics of empathy and schizotypy	Not tested	Positive associations with empathic concern and with positive schizotypy	See comments	[28]	Used metric of RHI sensitivity that combined subjective ownership measure with proprioceptive drift
Typical subjects scored for psychosis proneness	Not tested	Positive association with positive psychosis-like traits	No effects	[62]	No differences for negative schizotypal traits
Typical subjects scored for psychiatric vulnerability traits (with SCL-90)	Not tested	Positive associations with paranoid ideation, and psychoticism	No effects	[63]	Also found higher disownership of real hand positively associated with psychoticism, and higher ownership of rubber hand positively associated with interpersonal sensitivity
Schizophrenia subjects versus controls	Not tested	Higher in subjects with schizophrenia	Not tested	[64]	
Schizophrenia subjects versus controls	Faster in schizo-phrenia subjects	Decreased across trials in schizophrenia subjects, and increased in controls	Not tested	[65]	Schizophrenia group reportedly failed to learn the illusion as well as controls
Schizophrenia subjects versus controls, plus levels of schizotypy in controls	Not tested	Higher in subjects with schizophrenia; positively associated with schizotypy in controls	Higher in subjects with schizophrenia	[66]	Positive association in controls found for both positive and negative schizotypy; in schizophrenia subjects, illusion strength correlated with positive but not negative symptoms
Schizophrenia subjects versus controls	Not tested	‘Marginally higher’ (*P*=0.07) in subjects with schizo-phrenia for asynchronous condition only	No effects	[67]	Self-report ratings positively correlated with strength of delusions in schizophrenia subjects
Schizophrenia subjects versus controls	Not tested	Higher in subjects with schizophrenia for asynchronous condition only	No effects	[68]	Positive symptoms not correlated with indices of RHI among schizophrenia subjects; also tested associations with eating disorder scales and body dysmorphic disorder diagnosis, see table entry below
Subjects with c9orf72-mutation frontotemporal dementia (FTD) versus sporadic and MAPT FTD versus controls	Not tested	Higher in subjects with c9orf72-mutation fronto-temporal dementia than each other group	Not tested	[69]	C9orf72-mutation frontotemporal dementia overlaps in its symptoms with schizophrenia [49], and also usually involves eating-disorder symptoms [70–72]
Borderline personality disorder subjects versus recovered subjects versus controls	Not tested	Higher in borderline personality disorder subjects than in controls	No effect	[73]	Positive association also found between level of dissociation and illusion strength (ownership)
Psychosis-mimetic drug effects					
Effects of dexamphetamine versus controls	Not tested	Higher in dexamphetamine group	No effect	[74]	Dexamphetamine also induced loss of ownership of own hand
Effects of chronic ketamine use versus controls	Not tested	Higher in ketamine group	No effect	[75]	Chronic ketamine use positively associated with schizotypal and dissociative symptoms
Effects of ketamine administration versus controls	Not tested	Higher in ketamine group	Higher in ketamine group	[76]	
Eating disorders and body dysmorphic disorder					
Anorexia plus bulemia versus controls, and scored for eating disorder symptoms	Not tested	Higher in anorexia plus bulemia; positively associated with symptoms	Higher in anorexia plus bulemia	[77]	Participants include 36 with anorexia (24 restrictive, 12 binge/purge subtypes), 22 bulemia, 20 eating disorder not otherwise specified
Current eating disorder subjects versus recovered subjects versus controls	Not tested	Higher in eating disorder and recovered subjects than in controls	No effects	[78]	Same participant group as in [77] with addition of recovered eating disorders group (*N*=28)
Anorexia subjects versus controls	Not tested	Higher in anorexia subjects	No effect	[79]	Participants include 15 with anorexia nervosa, and 5 with eating disorder not otherwise specified
Typical subjects varying in scores for eating disorder symptoms	Not tested	Positively associated with bulemia and body development interest subscales	Not tested	[80]	Participants scored for three subscales of EDI-2 (Eating Disorder Inventory-2): bulemia, body dissatisfaction, and drive for thinness. Effects found for left hand, not right hand
Anorexia subjects versus controls	Not tested	Higher in anorexia subjects	Not tested	[81]	Participants with anorexia only; subtypes not discussed. Effects of illusion on hand reaching position also greater in anorexia subjects
Typical subjects scored for eating disorder symptoms	Not tested	No effects	No effects	[82]	Healthy participants scored for eating disorder questionnaire. Also tested effects of intranasal oxytocin; see table entry below
Subjects with Body Dysmorphic Disorder, or schizophrenia, and controls	Not tested	No effects of Body Dysmorphic Disorder diagnosis	No effects of body dysmorphic disorder diagnosis	[68]	Participants scored for three subscales of EDI-3 (Eating Disorder Inventory-3): bulemia, body dissatisfaction, and drive for thinness. Positive correlations of self-report illusion strength, but not proprioceptive drift, with these three eating disorder subscales across entire sample.
Oxytocin effects					
Typical subjects varying in serum oxytocin	Not tested	Higher in subjects with higher serum oxytocin	No effect	[60]	
Typical subjects given intranasal oxytocin versus placebo	Not tested	Higher in subjects given oxytocin	No effect	[82]	
Typical subjects given intranasal oxytocin versus placebo	Faster in subjects given oxytocin	Higher in subjects given oxytocin	Not tested	[83]	Illusion onset also slower for individuals with larger amygdala; amygdala said to ‘protect’ against the illusion; amygdala noted as larger in autism
Empathy and emotional intelligence					
Typical subjects scored for experience of pain of others	Not tested	No effect	Higher in subjects with stronger empathy for pain	[84]	
Typical subjects scored for experience of pain of others	Not tested	Higher in subjects with vicarious pain response	Not tested	[85]	
Typical subjects scored for empathy with Empathy Quotient (high versus low)	Not tested	Higher in high-empathy subjects	No effect	[86]	Positive association also found between strength of RHI (ownership and proprioceptive drift) and self-reported response to apparent pain induction in rubber hand
ASD subjects	No effect	No effect	Lower in subjects with lower empathy(by ADOS)	[57]	
Typical subjects scored for emotional intelligence (Mayer-Salovey-Caruso test)	Not tested	Positive association with emotional intelligence score	No effect	[87]	Emotional intelligence is positively correlated with empathy

Of the seven studies of eating disorders, four involved comparison of clinical groups, two focused on typical subjects scored for self-report eating disorder symptoms, and one [68] involved a mixture of clinical and control subjects (Table 1). Six of the seven studies showed that illusion strength was stronger among subjects with eating disorder diagnoses, or higher levels of symptoms, and one study of typical individuals found no differences. These findings are notable given the high psychological and psychiatric diversity found among subtypes of eating disorders, which is reflected and described in the studies included here (Table 1).

All three studies of oxytocin effects, one involving serum levels, and two based on intranasal administration, showed positive associations of this neuropeptide with illusion strength. All four studies of empathy (two for pain, one for self-report empathy (the Empathy Quotient), and one for ADOS-based empathy in autism subjects) also showed positive associations of these measures with strength of the illusion, as did self-report emotional intelligence (Table 1).

### 3.2. Associations among predictor variables

**Figure 2.**
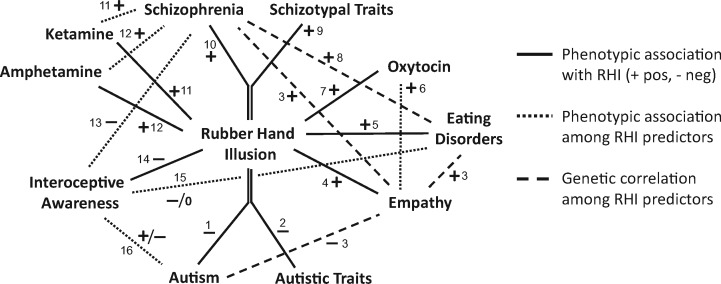
Relationships of rubber hand illusion susceptibility with autism spectrum and psychotic-affective spectrum disorders, and relationships among relevant predictor variables. Negative relationship shown as ‘−’, positive relationship as ‘+’, and no difference as ‘0’. Citations: 1 and 2: (Table 1); 3: [89]; 4–7: (Table 1); 8: [88, 89]; 9–12: (Table 1); 13: [90]; 14 (see text); 15: (negative: [91–94]; [95] (one condition); [96]; no difference: [97]; [95] (one condition); [98]; [99]); 16: (positive: [100] (one condition); negative: [101, 102]). Borderline personality disorder (one study) [73] is discussed in the text; this conditions showed no difference from controls in interoceptive accuracy (one study [103])

To minimize methodological heterogeneity in the genetic correlation estimates, the minimum number of sources (two) of information were used: Bulik-Sullivan et al. [88] for pairwise correlations between autism, anorexia and schizophrenia, and Warrier et al. [89] for the Empathy Quotient in relation to autism, anorexia and schizophrenia. As shown in Fig. 2, anorexia risk showed a significant positive genetic correlation with risk of schizophrenia, but neither anorexia and autism, nor schizophrenia with autism, showed significant genetic correlations with one another. Empathy Quotient scores were significantly positively genetically correlated with risk of schizophrenia, and risk of anorexia, but significantly negatively genetically correlated with risk of autism. Considered together, these two sets of findings show that schizophrenia risk, anorexia risk and Empathy Quotient scores are all positively genetically correlated with one another, but that autism risk is only genetically correlated, negatively, with Empathy Quotient score (Fig. 2).

The phenotypic relationships of autism, anorexia, and other psychotic-affective disorders with empathy (as measured by the EQ) are complex and, for simplicity of presentation, are discussed here rather than included in Fig. 2. Individuals with autism, and typical individuals scored for autism traits, have lower scores on the EQ, across numerous and diverse studies (e.g. [104]). These findings are in keeping with Baron Cohen’s theory of a central role for reduced empathy in autism.

For anorexia, Bremser and Gallup [105] found that EQ scores were positively associated with scores on the EAT-26 eating disorders scale, among typical individuals, in two separate populations. Of three studies that compared subjects with anorexia to controls for EQ score, two of them [106, 107] found no differences, and the third found no difference in a 16- to 18-year age group, but significantly lower empathy in anorexia subjects aged 12–15 years than in age-matched controls [108]. Further studies, especially of non-clinical individuals scored for anorexia traits, and for the EQ, are required for robust interpretation of these latter patterns.

Schizophrenia subjects show lower EQ than controls in three studies [109–111], and in a fourth study, they exhibit lower EQ when rated by relatives but not by self-report [112]. Studies of non-clinical schizotypy show a contrasting pattern of results, in that two studies show no differences on the EQ between subjects with high versus low schizotypy [113, 114], one study showed a negative association of EQ score with negative schizotypy, but no relationship for positive schizotypy [115], and one study showed that the cognitive empathy component of EQ was associated positively with positive schizotypy, but negatively with negative schizotypy, while the emotional empathy EQ component was not associated with positive schizotypy, and was associated negatively with negative schizotypy [116]. Taken together, these studies suggest that EQ scores are reduced in schizophrenia and negative schizotypy, but not in relation to positive schizotypy; further studies of these relationships are required for clear interpretation, and to investigate the causes of the positive genetic correlation of schizophrenia risk with EQ score.

Oxytocin administration or serum levels have been positively linked with a broad suite of measures of empathy (reviews in [55, 117]). For the EQ, oxytocin administration has been shown to increase measures of empathy for typical subjects with low EQ scores, but not for those with high EQ scores [118–120]. Studies to determine if oxytocin administration, or serum levels (baseline or responses to empathy-inducing stimuli), are linked with EQ would be useful to determine the nature of this association.

Interoceptive awareness, which is typically assessed using accuracy of heartbeat detection, is strongly inversely associated with strength of the rubber hand illusion [3, 52]. As such, it provides an ‘internal control’ on illusion strength in relation to its predictor variables. As predicted, heartbeat detection accuracy has been shown to be reduced, relative to controls, in most (7 of 11) studies of subjects with schizophrenia or eating disorders; it shows no significant difference from controls, however, in a single study of borderline personality subjects [103]. As also predicted, heartbeat detection has been shown as more-accurate in autism subjects, in one study [100], for subjects estimating it over longer time periods; in contrast for shorter periods in this study, and for other studies heartbeat detection was reduced in autism subjects (Fig. 2). Additional studies would be useful here, especially ones that tested for both interoceptive accuracy and illusion susceptibility in the same subjects.

## 4. DISCUSSION

The main results of this study are 2-fold. First, the rubber hand illusion shows consistent evidence of being reduced among individuals on the autism spectrum, and increased among individuals with schizophrenia or high levels of positive schizotypy. Depending on the study, these alterations involve some combination of changes in time required to feel the illusion, self-reported illusion strength, and perceived proprioceptive drift. The illusion was also increased among individuals with eating disorders or with borderline personality, two conditions also commonly considered as situated on the psychotic-affective spectrum, and after pharmacological treatment with the psychosis-inducing agents ketamine and amphetamine. On the psychotic-affective spectrum, rubber hand illusion effects also tend to be specific to cognitive-perceptual ‘positive’ symptoms, which involve manifestations of hallucinations and delusions; this pattern is not unexpected given that the illusion can be considered as a form of non-pathological hallucination.

Considered together, rubber hand illusion studies published to date thus support a diametric nature to rubber hand illusion susceptibility and strength in autism compared with psychotic-affective disorders. This hypothesis can be evaluated further by (i) including individuals on the autism spectrum, and the psychotic-affective spectrum, in the same rubber hand study, (ii) determining the degree to which the neurological and hormonal mechanisms of rubber hand effects also demonstrate diametric patterns, (iii) analysing in more detail the interpretation of verbal self-report, compared with proprioceptive drift based, measures of illusion presence and strength, and (iv) conducting studies of related bodily illusions, such as the full body illusion, in subjects with schizophrenia and autism; recent studies show that autism involves reduced effects of this illusion (as well as reduced peripersonal space) [121], though schizophrenia does not involve altered effects compared with controls [122]. Animal models should also be useful for additional tests, given the recent development of a ‘rubber tail illusion’ protocol in mice [123], for which autism-model knockout mice show reduced illusion effects compared with wild**-**types.

Support for the diametric model in this context suggests that embodiment**—**the felt and conceptualized self within the body**—**shows opposite deviations from typicality on the autism and psychotic-affective spectrums, in manners that correspond with descriptive and first-person accounts of sharper self**-**boundaries in autism, compared with more-malleable, porous self-other interfaces in schizophrenia [12, 16, 121, 124, 125]. These findings are important in extending the diametric model to embodiment and embodied cognition, and to our understanding of autism and psychosis with regard to Bayesian predictive coding, one of the most highly developed sets of models for how the human nervous system generates, perceives and processes information.

**Figure 3.**
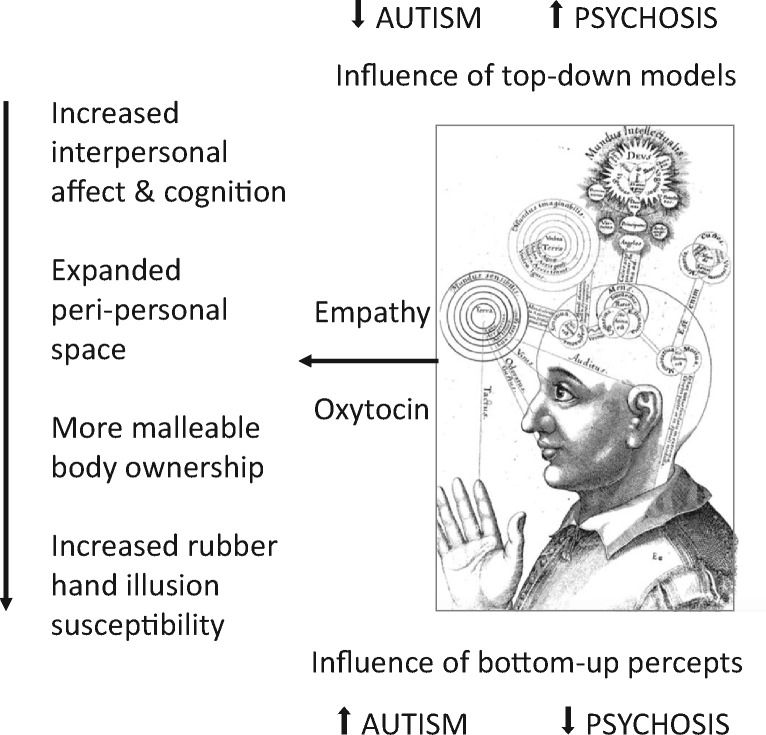
Framework, based in predictive coding, for interpretation of lower rubber hand illusion susceptibility in autism spectrum conditions, but higher susceptibility on the psychotic-affective spectrum. Drawing by Robert Fludd (1574–1637)

Reduced sensitivity to the rubber hand illusion on the autism spectrum has been attributed to effects from higher sensory precision and influence, such that tactile, proprioceptive and interoceptive input from the true hand is less-readily overridden by visual input from the false hand being touched [57, 84, 126] (Figs 1 and 3). This hypothesis is consistent with considerable evidence of sensory enhancements among individuals on the autism spectrum [127, 128], which may extend to interoceptive and proprioceptive sensation [129], as well as with higher somatosensory sensitivity in primary sensory cortex for auditory, visual and tactile stimulation [130–132]. No studies have tested for associations between perceptual acuities or sensitivities and rubber hand illusion susceptibility, in autism spectrum or typical subjects; however, the only other group of subjects known to exhibit significantly reduced susceptibility to the illusion is expert pianists [133], who might be expected to exhibit enhanced proprioceptive skills.

Non-exclusively to a hypothesis based on sensory enhancements, reduced illusion sensitivity on the autism spectrum may also be due, in the Bayesian predictive coding framework, to reduced effects of higher-level, top-down, a priori frameworks, such that sensory evidence is afforded relatively more weight in the detection and processing of prediction errors [41, 134, 135] (Fig. 3). Reduced high-level, top-down model precision can be regarded as corresponding to weak central coherence in autism, one of its main explanatory psychological frameworks [136].

Reduced illusion susceptibility on the autism spectrum may additionally be associated with lower empathy, given positive linkages between measures of empathy (including the Empathy Quotient) and illusion susceptibility, and positive associations of oxytocin levels with both empathy and the illusion. Higher levels of autism-related sensory symptoms have also been associated with lower empathy by the Empathy Quotient [137] (see also [138]). These results are intriguing in that they link embodiment with sensory sensitivity and cognitive-affective connectivity of self to others; they thus connect the social-empathic domain of autism with the domain of repetitive behaviors and restricted interests, given strong associations of repetitive behavior with sensory phenomena [139]. Like empathy, higher emotional intelligence has also been associated with stronger rubber hand illusion susceptibility [140], and emotional intelligence is reduced in autism [141, 142].

Enhanced rubber hand illusion sensitivity on the psychotic-affective spectrum can been understood in the context of these conditions involving perception that is less dependent on sensory input, and more sensitive to higher-level beliefs, as in most predictive coding accounts of psychosis [44, 46, 135, 143, 144] (Fig. 3). By this logic, the illusion induces a relatively strong prediction error in the discordances between vision and the other senses, which is reduced (minimized) by an updated delusional top-down belief that the rubber hand is one’s own. Schizophrenia indeed involves reduced sensory acuity [145, 146] and such sensory deficits are positively associated with hallucinations and delusions [144, 145, 147, 148]. Consistent with this interpretation, congenital blindness protects against the development of schizophrenia, apparently due to compensatory enhancements of other senses [147, 149]. A role for sensory and multisensory-integration deficits in greater rubber hand illusion effects in schizophrenia and other psychotic-affective conditions, compared with autism, is supported by several additional lines of evidence, including:

- a link between reduced tactile sensitivity and higher susceptibility to the ‘Pinocchio’ somatosensory illusion in schizophrenia subjects [150];
- reduced effects of proprioceptive signals as a postulated cause of the increased rubber hand illusion intensity among individuals with anorexia [81] and reduced intensity in autism [57, 151];
- increased reliance on proprioception compared with other modalities [126], as well as enhanced proprioceptive learning [152] (see also [134]) in autism spectrum subjects compared with controls, but impaired proprioception in schizophrenia subjects, which is linked to self-disorder intensity [153] and
- evidence for enhanced early-sensory processing in autism as detected by mismatch negativity (cortical signals of auditory deviance detection, interpretable as prediction error sensing) [154, 155]. In contrast, mismatch negativity is strongly and highly consistently reduced in schizophrenia [156, 157].

In contrast to hallucinations mediated by bottom-up perceptual processes, increased effects from top-down cognition in psychotic-affective conditions are expected to involve delusional and other beliefs overly strongly held for the evidence supporting them, including those derived from jumping-to-conclusions [158]. Such top-down effects may follow in part from learning (and other cognitive) deficits [159] that are broadly symptomatic of the psychotic-affective spectrum.

Most generally, whatever alterations to predictive coding processes mediate rubber hand illusion susceptibility in autism, compared with schizophrenia and other psychotic-affective spectrum disorders, they presumably, overall, have opposite effects to one another, to account for the patterns documented here (Fig. 3). Opposite predictive-coding alterations, in autism compared with psychosis, have been proposed by several authors [135, 143].

The second main result of this study is that the psychiatric correlates of the rubber hand illusion analysed here can be situated within a causal network of phenotypic associations and genetic correlations that is highly internally consistent and consilient, in that multiple independent lines of evidence show convergent results (Fig. 2). These results include:

- all-positive pairwise genetic correlations between schizophrenia genetic risk, anorexia genetic risk, and empathy score (self-report Empathy Quotient), which indicate overlapping genetic bases for these three traits; all three also involve increased rubber hand susceptibility. In contrast, autism genetic risk is negatively genetically correlated with empathy score, and shows reduced illusion susceptibility;
- the positive associations of oxytocin with a stronger illusion, and myriad studies linking higher oxytocin to higher empathy (by different measures, including the EQ) and
- generally reduced interoceptive awareness (as measured by heartbeat detection) in schizophrenia and in some studies of eating disorders, and mixed results in autism; lower interoceptive awareness is generally associated with higher illusion susceptibility.

The findings that schizophrenia, anorexia and empathy are all positively associated at the genetic level indicates that some alleles pleiotropically affect both empathy and schizophrenia risk or eating disorders risk, and increase risk of both schizophrenia and anorexia. At the phenotypic level, eating disorder questionnaire scores, in healthy populations, have been positively associated with both Empathy Quotient scores and scores for positive schizotypy [105]. The main inconsistencies in these findings are that EQ scores are lower among subjects with schizophrenia than in controls, that schizotypy among typical individuals shows a mixed relationship with EQ scores, and that some authors interpret social deficits found in anorexia as evidence of autism spectrum traits (e.g. [108]). For schizophrenia, deleterious environmental effects during development presumably prevent the positive genetic correlation with empathy from manifesting as a positive phenotypic one; additional studies are needed to understand schizotypy, especially positive schizotypy, in relation to the EQ in particular and empathy in general. Finally, empathy (by the EQ), non-clinical positive schizotypy, and eating disorder prevalence, as well as borderline personality disorder prevalence, all show strong female gender biases [104, 160–163], which fit with the connections between them in terms of sex-differential patterns and liabilities.

How can these consilient findings be interpreted, especially in light of the links of schizophrenia, anorexia and empathy with increased susceptibility to the rubber hand illusion? Are the self, and self-other relationships, altered in similar ways among individuals with high empathy, positive schizotypal traits or schizophrenia, and eating disorders, and presumably also borderline personality?

In contrast to reduced empathy and self-to-other links on the autism spectrum, self-perception, self-other connections and interpersonal sensitivities can be seen as increased, in negative and psychologically problematic ways, in these three overlapping psychotic-affective conditions. Thus:

- Schizophrenia and positive schizotypy are characterized by (i) paranoia, persecutory delusions, and negatively valenced auditory hallucinations, as well as (ii) the Schneiderian self-disorders including thought withdrawal, insertion or broadcast, all of which directly reflect forms of self-other interaction [11] and (iii) altered perception of body image [164]. This interpretation also fits with (iv) enhanced mirror system activation among actively psychotic individuals with schizophrenia, in contrast to reduced activity, for the same protocol, in autism [165], and (v) higher activity of the right temporal parietal junction, a region that subserves self-other representations [166], self-location and perspective-taking [167, 168] and perception of the rubber hand illusion [169], in schizophrenia [170, 171], in comparison to lower activity of this region in autism [172, 173], and (vi) greater overlap between brain areas involved in self, compared with other, neural processing [174], as well as higher perceived similarities between self and others [175], in schizophrenia subjects than in controls.
- Anorexia involves (i) a self-observing, critical ‘anorexic voice’, comparable in negative and pejorative context to the running-commentary form of auditory hallucinations [176], (ii) highly altered perception and images of one’s body (e.g. [177]); (iii) increased anxiety regarding negative evaluation and rejection by others [105], (iv) high levels of positive schizotypy, including suspiciousness and delusions [105, 178], and (v) internalized interpersonal sensitivities involving increased self-reflectivity, high levels of shame, and low self-esteem [179]. Sours ([180], p. 343) indeed stated that in anorexia ‘their primary disturbance is the perception of the self, not simply that of the body’. Finally, anorexia and schizophrenia overlap in incidence and diverse phenotypes [181].
- Borderline personality disorder is characterized by (i) fear of social rejection or abandonment, (ii) intense and unstable interpersonal relationships, (iii) suspiciousness and paranoia, and stress-induced psychotic episodes [182], (iv) feelings of emptiness and fear of having one’s identity and self ‘taken over’ by other individuals [183, 184] and (v) high levels of empathy [185]. In this context, the self is ‘impoverished, poorly developed, or there is an unstable self-image, which is often associated with excessive self-criticism; chronic feelings of emptiness; and dissociative states under stress’ [186]. This disorder was originally created for persons at the ‘border’ of psychosis with neurosis (mainly depression and anxiety) (see [187]); it is also highly comorbid with eating disorders including anorexia [188, 189], and weight preoccupation can be predicted from borderline personality traits [190].

Schizophrenia, anorexia, and borderline personality are also each strongly mediated by childhood trauma and emotional or physical abuse, and involve dissociation, the disconnecting from one’s thoughts, feelings and identity, as one of their sequelae [73, 191–195].

How might empathy and high interpersonal sensitivities, in both their positive and negative manifestations, contribute to higher rubber hand illusion sensitivity in psychotic-affective conditions? The simplest explanation is that these conditions engender high awareness of, and sensitivities to, others in relation to the self, in the context of more porous and malleable self-other boundaries [40] (Fig. 3). Such weaker boundaries then manifest as easier transfer of the self**-**hand to the rubber hand. Sensory and multisensory integration deficits, especially in interoception and proprioception, presumably facilitate this transfer by reducing the strength of one’s self-body ownership. Oxytocin, in turn, contributes to the process, and to illusion sensitivity, though its effects on promoting a salience-system mediated, and insula mediated, switch from internally to externally social-directed attention and cognition [54, 196]. This hypothesis is consistent with decreases in interoceptive accuracy caused by oxytocin [196], and with reduced oxytocin effects among individuals with autism spectrum conditions [53], but increased and dysregulated oxytocin-system effects in relation to psychotic-affective symptoms and conditions, especially among females [55]. As such, trade-offs between internally and socially externally directed attention and salience may be oppositely affected, and extreme, in these two sets of conditions.

The findings described here have implications in diverse clinical contexts, including stroke rehabilitation, adjustment to limb loss, neglect, anosognosia (unawareness of some disability) and asomatognosia (denied ownership of a body part), in that positions of individuals along an axis from autism to typicality to psychotic-affective conditions should impact upon the efficacy and mechanisms of embodiment, and in that, as suggested, sensory acuities may strongly mediate these processes. The results also support the development of novel perspectives and therapies for autism, schizophrenia, and anorexia, that are based on experimental alterations to perception, self-perception and self-other boundaries [40, 197–199]; for example, the internal ‘emptiness’ expressed by many subjects with borderline personality and schizophrenia [184, 187] may be due to reduced efficacy and cognitive influence of interoception [33] that can be ameliorated by training [200]. Similarly, the mechanisms that ‘protect’ autism spectrum individuals from the psychotic nature of the rubber hand illusion (including amygdala effects [83]) may be useful for treatment of individuals with psychosis, or those at high risk. Therapies for eating disorders that address forms of excessive empathy, self-reflectivity, and psychotic-affective cognition may be especially useful; in the longer term, determining what alleles overlap between anorexia, empathy and schizophrenia, apparently giving rise to the observed positive genetic correlations between then, would be uniquely informative.

The main limitations of this review are that sufficient and appropriate information are not yet available for a thorough meta-analytic approach to the questions addressed, and that rubber hand illusion methodologies vary between studies in ways that may influence the outcomes. Moreover, no studies have collected the joint set of data, on illusion susceptibility, interoceptive accuracy, proprioceptive (and other sensory) acuity, and empathy, that would be most-directly useful in hypothesis testing. Corollary feedback strength, which is lower in schizophrenia (e.g. review in [144]), may also play a central role in rubber hand illusion effects; it can be predicted to be higher or preserved on the autism spectrum, but has apparently not been studied in this context. Eating disorders are also highly heterogeneous, and would benefit from targeted study of the rubber hand illusion and predictive coding in subjects homogeneous for their subtypes, rather than in pooled samples.

The conceptual basis of this review is also centered on predictive coding; the rubber hand illusion can also be considered in terms of multisensory integration of visual and tactile signals, which exhibits extended temporal binding windows, compared with controls, in both autism and schizophrenia [201]. The similarity of these alterations suggests that they are unlikely to mediate opposite patterns of rubber hand illusion effects, unless they are caused by divergent neurophysiological effects [125]. More broadly, study of associations of the embodied self with psychological problems needs to focus not simply on psychiatric diagnoses, but on the adaptive psychological and neurological mechanisms that underpin them, and how their variation in opposite directions can, at relative extremes, lead to maladaptive and diametrical traits.

A final potential limitation is that most rubber hand illusion susceptibility data comes from subjective self-reports (for onset time and intensity) rather than from information on the magnitude of proprioceptive drift, which could be considered as more objective and quantifiable; in principle, subjects with schizophrenia may thus be more ‘suggestible’ with regard to self-reporting RHI effects. In Table 1, support from both self-report and proprioceptive drift is found in each the four categories for autism, psychotic-affective disorders, drug effects, and empathy effects, though to variable degrees; further studies are needed on neurophysiological meanings of the metrics of the RHI, and their inter-relationships.

Anaïs Nin has been retrospectively ‘diagnosed’ with schizotypal and borderline traits, high levels of dissociation, and various Axis 1 phenotypes, which may underlie her creative, insightful writings [202]. Her belief, that we see the world as we are, rather than as it is in some objectifiable way, is exemplified by embodied cognition, its illusions, and the psychiatric conditions with which it associates. The question then becomes how to become who we wish to be, through better understanding and manipulation of the self. As Nin opined in a 1972 interview: ‘I think compassion takes empathy, and empathy takes imagination. You have to be able to displace yourself to put yourself in the place of another’.
